# Neural coding of intended and executed grasp force in macaque areas AIP, F5, and M1

**DOI:** 10.1038/s41598-018-35488-z

**Published:** 2018-12-20

**Authors:** Rijk W. Intveld, Benjamin Dann, Jonathan A. Michaels, Hansjörg Scherberger

**Affiliations:** 1 Deutsches Primatenzentrum GmbH, Kellnerweg 4, 37077 Göttingen, Germany; 2 Department of Electrical Engineering, Stanford University, Stanford, CA 94305 USA; 3 Faculty of Biology and Psychology, University of Goettingen, 37073 Göttingen, Germany

## Abstract

Considerable progress has been made over the last decades in characterizing the neural coding of hand shape, but grasp force has been largely ignored. We trained two macaque monkeys (*Macaca mulatta)* on a delayed grasping task where grip type and grip force were instructed. Neural population activity was recorded from areas relevant for grasp planning and execution: the anterior intraparietal area (AIP), F5 of the ventral premotor cortex, and the hand area of the primary motor cortex (M1). Grasp force was strongly encoded by neural populations of all three areas, thereby demonstrating for the first time the coding of grasp force in single- and multi-units of AIP. Neural coding of intended grasp force was most strongly represented in area F5. In addition to tuning analysis, a dimensionality reduction method revealed low-dimensional responses to grip type and grip force. Additionally, this method revealed a high correlation between latent variables of the neural population representing grasp force and the corresponding latent variables of electromyographic forearm muscle activity. Our results therefore suggest an important role of the cortical areas AIP, F5, and M1 in coding grasp force during movement execution as well as of F5 for coding intended grasp force.

## Introduction

In everyday life, we grasp many objects that vary in shape, size, and weight. For planning the appropriate hand action, the brain predominantly uses visual information to estimate the required reach direction, hand shape, and grasp force. It then generates a motor plan to accurately generate the required muscle activation. The brain achieves this task remarkably well, but the exact mechanisms behind this process, including the planning and control of grasp force, are still unclear.

Studies investigating voluntary grasping actions have focused on the cortical planning and execution of hand shape in three cortical areas, the hand area of primary motor cortex (M1)^1–5^, ventral premotor cortex (area F5)^6,7^, and the anterior intraparietal area (AIP)^8,9^. In each of these areas, neurons can be found that are selective for different grasp movements. Furthermore, neural activity in AIP and F5 is modulated by the observed object or grasp instruction before movement execution, which is believed to play a role in grip type planning^7,8,10–15^.

Since neurons in AIP, F5, and M1 play an important role for the control of hand shape, one could expect them to be highly relevant for the control of grasp force as well. Indeed, representation of grasp force has been demonstrated for single-units in M1^16–21^. However, only one study showed a weak force coding in area F5^22^ and, to our knowledge, no such study has been conducted in AIP and none has investigated the encoding of *intended* grasp force, i.e., before the movement, in any of these areas.

Due to technological advancements, simultaneous recordings from many neurons are now possible. This allows us to draw conclusions from population responses of many, simultaneously recorded neurons by means of dimensionality reduction^10,23^. Such an approach has recently demonstrated strong similarities in the temporal activation of the M1 population across task-dependent as well as task-independent muscle representations in M1^24^. This leads to the question how well these representations are preserved across the key areas for grasp planning in the fronto-parietal grasping network, i.e., AIP and F5.

To address these issues, we trained two macaque monkeys to perform a delayed grasping task where a handle had to be grasped with two different grip types and three different force levels. Single- and multi-units were recorded from a high number of channels in parallel in AIP, F5, and M1, which allowed us to directly compare grasp force representation between areas.

We found grasp force strongly encoded in all three areas, but intended grasp force, i.e., prior to movement, was mainly found in F5. To our knowledge, we demonstrate for the first time the representation of grasp force in single- and multi-units of AIP. Neural tuning patterns representing grip type and grasp force were often complex, heterogeneous, and difficult to interpret. However, dimensionality reduction methods revealed a low-dimensional population structure that captured most of the task-specific variance. Intriguingly, latent variables representing the common temporal reach to grasp related activity and dimensions representing the temporal grasp force related activity were highly correlated with the corresponding part of forearm muscle activity. These results suggest a remarkable direct control of grasp force by neural populations of the fronto-parietal grasping network.

## Results

### Behavior

**Figure 1:**
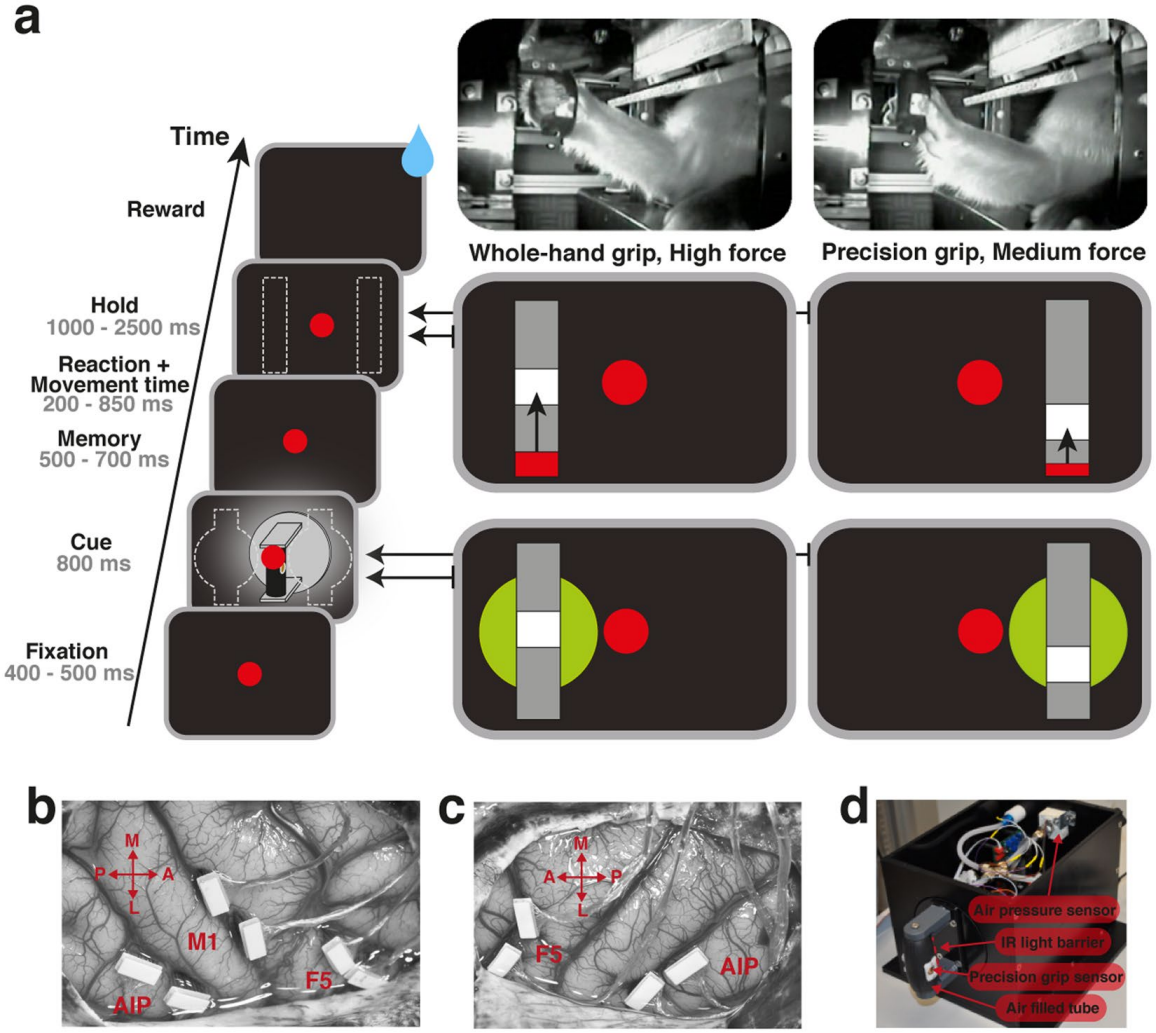
Task paradigm and recording locations. (**a**) Task paradigm. Each rectangle on the left shows the image visible during one epoch. Specific cue and hold epochs of two example conditions are shown on the right. Red slider in the hold epoch provides online feedback of the currently applied force while the white square indicates the required amount of force. Arrows illustrate where the red slider had to move, but were not visible during the task. Pictures on the top show monkey S performing a whole-hand and a precision grip. (**b**) Picture of arrays implanted in the right hemisphere of Monkey B. From left to right: arrays implanted in AIP, M1, and F5. (**c**) Picture of arrays implanted in the left hemisphere of Monkey S. From left to right: arrays implanted in F5 and AIP. Arrows indicate the medial (M), lateral (L), anterior (A), and posterior (P) direction. (**d**) Picture of the force sensing handle. Lid of the box was removed for the picture to provide extra insight in its design. The handle would detect a precision grip when both touch sensors were touched and a whole-hand grip when the infrared light barrier in the handle opening was broken. Force was detected by measuring the change in air pressure inside the handle.

To investigate neural grasp force modulation in the frontoparietal grasping network, two monkeys were trained to perform a delayed grasping task, in which they grasped a handle either with a precision grip or whole-hand grip with different amounts of force (Fig. 1a,c). The instructed amount of force was successfully applied for a duration of 1 s in 95% of all executed trials in monkey B (11 sessions, on average 639.8 trials per session) and in 93% of all executed trials in monkey S (5 sessions, on average 476.4 trials per session), see Table 1. Grasp performance was high and similar for all conditions for both monkeys. However, the percentage of eye fixation errors before movement onset was much increased in monkey B, which we believe represents a lack of motivation, not of capability, to complete the task for that condition.

**Table 1:** Task performance. Percentage of successful trials (performance) and percentage of trials when eye fixation was broken (eye fixation errors) for monkey B and monkey S for the whole-hand low force (WLF), whole-hand medium force (WMF), whole-hand high force (WHF), and for the precision low force (PLF), precision medium force (PMF), precision high force (PHF) condition.

Task condition		WLF	WMF	WHF	PLF	PMF	PHF
Monkey B	Performance	98%	99%	95%	96%	93%	89%
	Eye fixation errors	33%	37%	36%	46%	61%	67%
Monkey S	Performance	96%	96%	90%	89%	96%	90%
	Eye fixation errors	5%	17%	26%	3%	4%	5%

To compare the task behavior of both monkeys, we calculated the reaction time, movement time, and acquisition time (see Methods). Reaction time was 268 ± 69 ms (mean ± SD) for monkey B and 257 ± 72 ms for monkey S. As expected, movement times differed significantly (Wilcoxon test, p < 0.001) between grip types in monkey S, with a movement time of 121 ± 14 ms for whole-hand grips and 192 ± 43 ms for precision grips. Movement times of monkey B were longer than for monkey S (p < 0.001) and surprisingly similar for whole-hand grips (216 ± 32 ms) and precision grips (220 ± 42 ms). Acquisition times were similar for both monkeys but depended on the force level, with lower force levels acquired earlier than higher force levels. For monkey B, average acquisition time was 113 ± 230 ms, 467 ± 295 ms, and 555 ± 381 ms for whole-hand low force (WLF), whole-hand medium force (WMF), and whole-hand high force (WHF) conditions, respectively, and 85 ± 215 ms, 561 ± 329 ms, and 718 ± 360 ms for precision low force (PLF), precision medium force (PMF), and precision high force (PHF) conditions, respectively. For monkey S, acquisition time was 11 ± 14 ms, 403 ± 304 ms, 622 ± 318 ms for WLF, WMF, and WHF conditions and 151 ± 312 ms, 385 ± 251 ms, 539 ± 332 ms for PLF, PMF, and PHF conditions, respectively. Altogether, we did not observe large differences in behavior, so that neural data of the two animals can be compared with confidence. The increasing acquisition time for the three force levels from low to high force was to be expected, since animals had to build up the required force, which is, in consequence, fastest for the low force and slowest for the high force condition.

**Figure 2:**
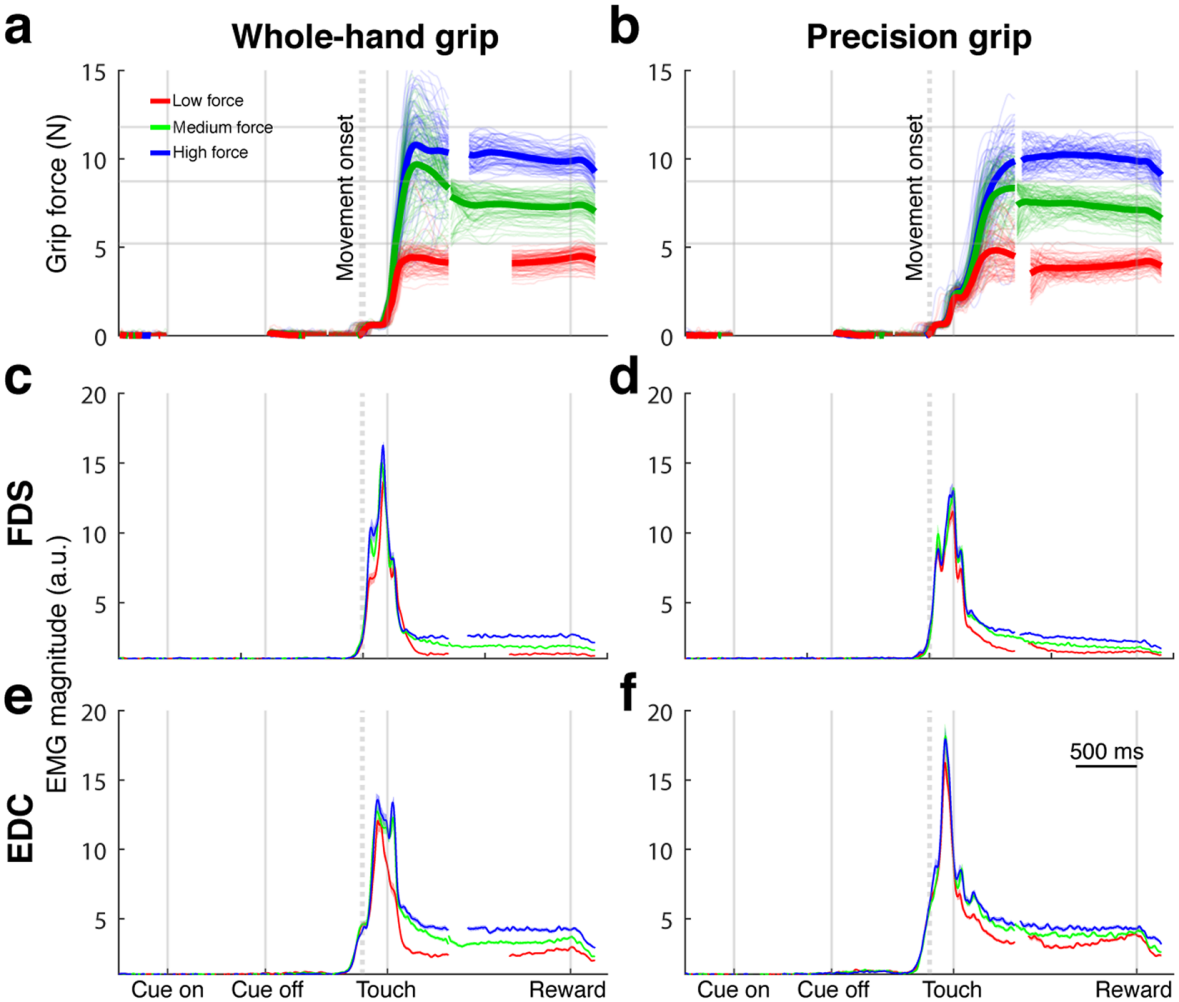
Force profile and EMG signals. Panels a and b show the amount of force applied over time for individual trials (thin transparent lines) and on average per condition (thick line) of a single session of monkey B (session Bt150219, 447 trials). Grey horizontal lines show the boundaries of the different force conditions corresponding to 5, 9, and 12 N. Low, medium, and high force conditions are shown in red, green, and blue, respectively. Note that the small deflection of grip force during and shortly after movement onset and before touch is due to a voltage change of the power supply caused by the release of the handrest button. Panels c and d show mean EMG signals of the FDS muscle over time (shaded area: standard error of the mean; very small in size). Panels e and f: same as c, d, but for EDC muscle. Left panels show whole-hand grips, right panels precision grips. Force and EMG signals are not shown for overlapping periods between touch and reward alignments (see Methods). Solid vertical lines indicate the alignments: cue onset, cue offset, touch, and reward. Dotted vertical lines indicate median movement onset before touch (whole-hand grip: 206 ms; precision grip: 197 ms).

Figure 2 illustrates the temporal force profile and the corresponding electromyogram (EMG) of the *flexor digitorum superficialis* (FDS) and the *extensor digitorum communis* (EDC) muscle in the different grasp conditions of an example session of monkey B. EMG magnitude peaked around touch (Fig. 2c–f), while the applied force reached its peak value somewhat later (Fig. 2a,b). As expected, higher grasp forces corresponded with higher EMG magnitudes. Importantly, no increase in EMG magnitude was observed before the Go Signal was presented, demonstrating that the monkey did not move its arm prematurely, i.e., before the movement start was indicated.

Already in the first 100 ms after touching the handle, force signals started to differ between conditions for both whole-hand grip (Fig. 2a): low force (WLF) vs. medium force (WMF) (Tukey-Kramer test, p < 0.001), low force (WLF) vs. high force (WHF) (p < 0.001) and precision grip (Fig. 2b): PLF vs. PMF (p < 0.001) and PLF vs. PHF (p < 0.001). However, the medium force and high force condition did not significantly differ in the first 100 ms after touch, neither for the whole-hand grip (p = 0.77), nor precision grip (p = 0.99). This early divergence of the low force condition from the medium and high force conditions strongly suggests that the monkey planned in advance whether or not to apply a low force. However, because it takes more time for medium and high forces to diverge, it is not possible to conclude from this data whether the distinction between these conditions was planned in advance or at a later time point based on continuous feedback.

This force pattern was generally observed in monkey B. In 9 out of 11 sessions in monkey B, we found a significant difference between the PLF and PMF/PHF condition. The two sessions where this was not the case were early in the recording stage. PMF and PHF did not significantly differ in any of these sessions. In monkey S, force levels were stored and analyzed only in one session, however with similar findings: in the first 100 ms after touch, force levels were significantly different between all conditions (p < 0.01), except for the WMF vs. WHF condition (p = 0.19).

In most trials the monkeys did not apply more force than required for that condition. However, overshoots did occur (see individual trials in Fig. 2a and b). Monkey B applied more force than required before reward onset in 22%, 68%, 34%, 14%, 48%, and 34% of all WLF, WMF, WHF, PLF, PMF, and PHF trials, respectively, whereas in monkey S it was 0%, 11%, 23%, 4%, 4%, and 20% of all WLF, WMF, WHF, PLF, PMF, and PHF trials, respectively. Together, monkey B applied more force than required most often for medium force conditions, while monkey S did so most often for the high force conditions.

### Neural responses of single-units

From two 32-channel electrode arrays (FMAs, see Supplementary Information) in each area (Fig. 1b), we recorded in monkey B on average 29.1 ± 5.6 units in AIP (mean ± SD), 46.3 ± 9.7 in F5, and 51.7 ± 7.1 units in M1. In monkey S, we recorded 67.0 ± 12.5 units in AIP and 63.2 ± 10.5 units in F5. Of these units, we characterized on average 11.2, 14.9, and 18.0 as single-units in each area of monkey B, and 24.0 and 29.6 in each area of monkey S (see Supplementary Information).

**Figure 3:**
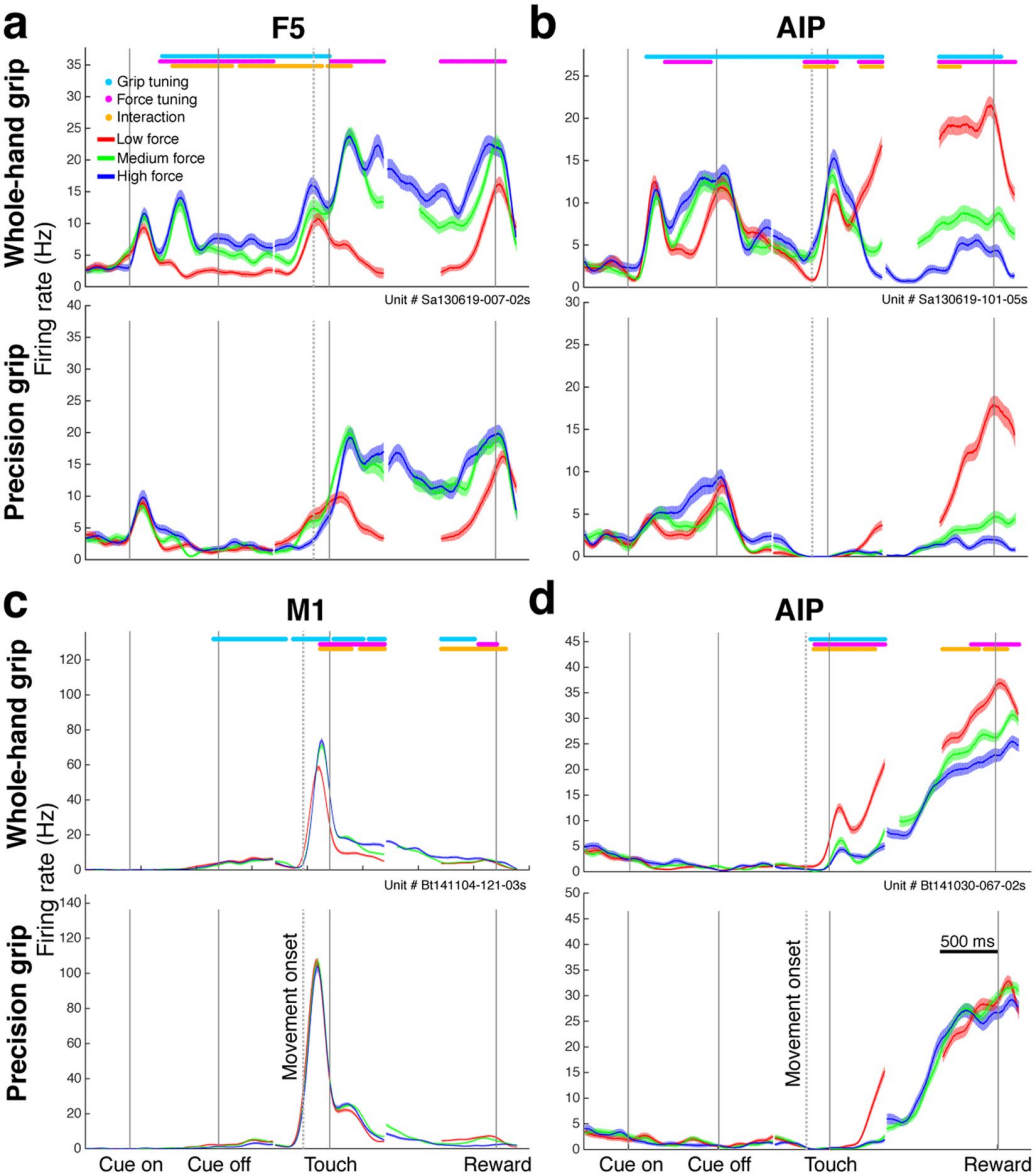
Single neuron responses. Convolved average firing rates of four example neurons (**a**–**d**) over time for low force (red), medium force (green), and high force (blue) conditions for whole-hand grip (top of panel) and precision grip trials (bottom of panel). Shaded areas: standard error of the mean. Colored horizontal lines on top of each panel (tuning lines) indicate time intervals with significant difference in firing rate between grip types (cyan), force (magenta), or with significant interaction (yellow) between grip type and force (cluster-based permutation test, p < 0.01). X-axis, alignments, epochs, and scale as in Fig. 2. (**a**) Example neuron from F5 of monkey S (session Sa130619, 419 trials). (**b**) Example neuron from AIP of monkey S (session Sa130619, 419 trials). (**c**) Example neuron from M1 of monkey B (session Bt141104, 498 trials). (**d**) Example neuron from AIP of monkey B (session Bt141030, 581 trials).

In each cortical area, we found a great variety of multi- and single-unit responses to the different task conditions. Figure 3 shows the peristimulus time histogram (PSTH) of four single-units recorded in F5 and AIP of monkey S (Fig. 3a,b) and M1 and AIP of monkey B (Fig. 3c,d). Significant firing rate differences (cluster-based permutation test, p < 0.01, see Methods) between grip types (grip tuning), force conditions (force tuning), or interaction are visualized as horizontal colored bars on top of the panels. Each type of tuning was observed at various time epochs, illustrating the variety of neural responses that we observed in these brain areas.

Significant firing rate differences between force conditions were also present in the cue and memory epochs, prior to movement (Fig. 3a,b). These differences could reflect the planning of different force levels. In accordance with the measured force and EMG signals (Fig. 2), we also observed an earlier divergence in the neural activity for the low force conditions compared to the divergence between medium and high force (see for example Fig. 3a–d).

Similar to previous studies^25,26^, we found units that were tuned for grip type before (Fig. 3A–C) and after movement onset (Fig. 3b–d). Furthermore, all example units showed significant interaction tuning for grip type and force at different moments of the task. These example units provide a first insight, but to better understand the role of these cortical areas for planning and controlling grasp force, we need to quantify the tuning for grip type, force, and interaction in the respective neuronal populations.

### Population tuning

**Figure 4:**
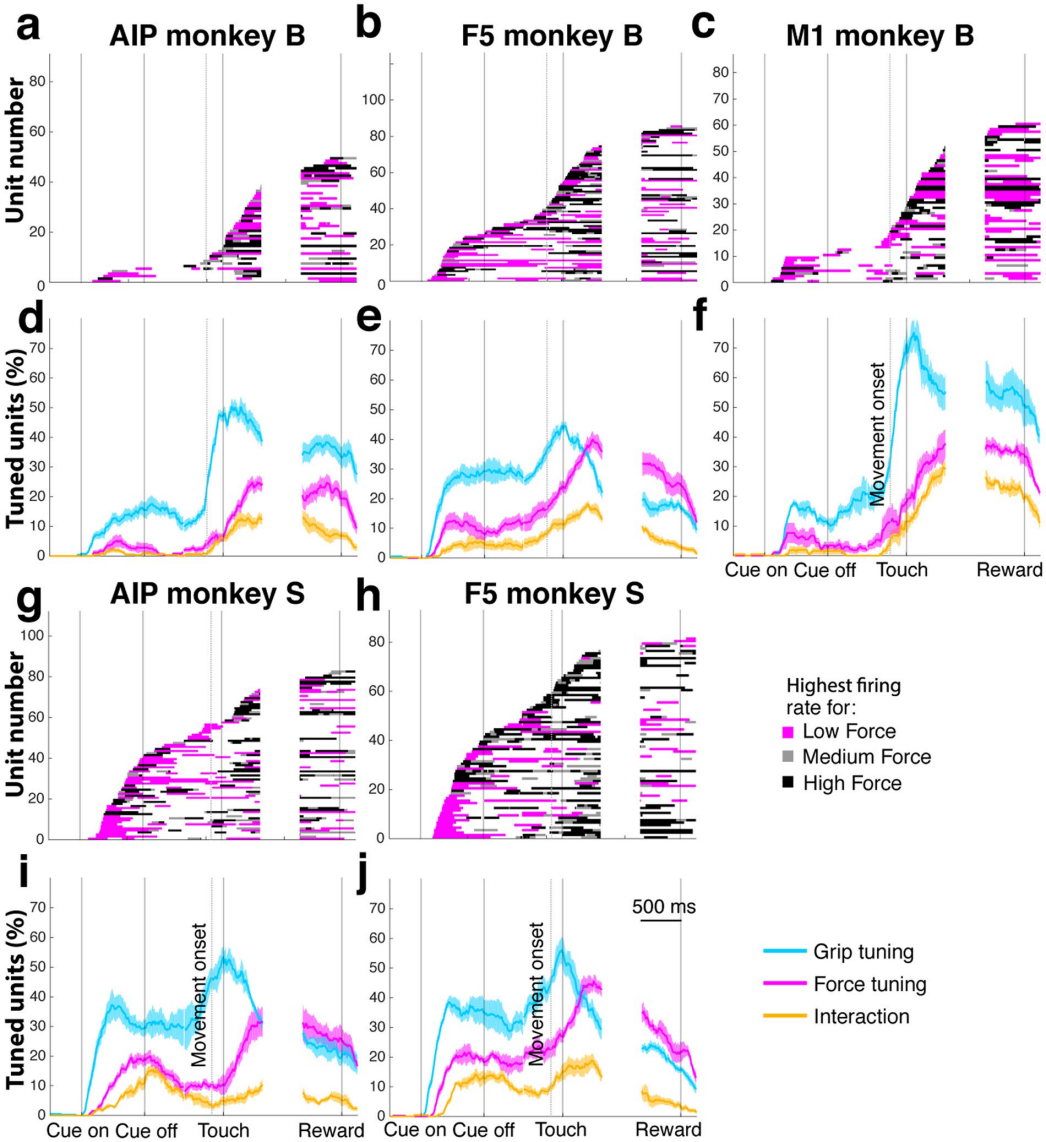
Population tuning of grip type, force, and their interaction. Panels a–c,g,h show force tuning lines, vertically sorted in ascending order by tuning onset, for the best-channel set of AIP (**a**), F5 (**b**), and M1 (**c**) of monkey B and of AIP (**g**) and F5 (**h**) of monkey S. Horizontal lines represent time intervals when a unit was significantly tuned for grip force, with the line color indicating whether the unit’s firing rate was maximal for the low (magenta), medium (grey), or high force condition (black). Y-axis: unit number in dataset. Panels d–f, (**i**,**j**) show the percentage of units at a particular time of the task with a significant tuning for grip type (cyan), force (magenta), or with a significant interaction effect (yellow) across all recorded sessions. Shaded areas: standard error of the mean across datasets. X-axis, alignments, epochs and scale as in Fig. 2.

To gain insight in the tuning responses across the population, we plotted time intervals with significant force tuning of the single- and multi-units of the best-channel set (see Supplementary Information), sorted by their tuning onset (Fig. 4a–c,g,h). For monkey B and S, 49/91 and 82/112 units of AIP were tuned for force for some duration of the task. Similar proportions were observed for F5 (85/126) and M1 (60/87) of monkey B and F5 of monkey S (81/93). To our knowledge, we show for the first time strong force tuning responses of AIP single- and multi-units, suggesting that this area is potentially involved in the control of grasp force. In addition, we confirm previous findings of force tuning in F5 and M1 single-units^17,18,20–22,27^.

The large heterogeneity of tuning onset/offset and tuning duration (Fig. 4a–c,g,h) makes it difficult to compare the temporal responses between the brain areas. We therefore show in addition in Fig. 4d–f,i,j the percentage of units tuned at every time point for force (magenta), grip type (cyan), and interaction effect (yellow).

For AIP (Fig. 4d,i) we see that both monkeys had between 20 and 30% of units tuned for force during the hold epoch. However, the amount of tuning in the cue and memory epoch was only substantial for monkey S (10–20%), but not for monkey B (<5%). A role of AIP in grasp force control seems therefore likely during grasp execution, but its role for grasp force planning remains unclear due to these differences between both animals.

Many more AIP units were tuned for grip type than for force, reaching percentages of 48% for monkey B and 51% of grip tuned units for monkey S at the time of object touch. Only in the last 500 ms of the hold epoch in monkey S (Fig. 4i) the percentages of force tuned units and grip tuned units were similar. We can also see that the steep increase in the percentage of grip tuned units preceded the steep increase of force tuned units in both animals. A possible explanation for this different time course could be that preshaping of the hand precedes the application of grip force. Percentages of units with a significant interaction effect show a similar pattern as for force tuning, but with lower percentages. This suggests that grip force might in part be coded independently from grip type, since only a smaller fraction of units showed an interaction effect.

F5 responses were similar to those observed in AIP (Fig. 4b,e,h,j), but the occurance of force tuned units was higher by roughly 10 percentage points in the late memory and early hold epoch for both monkeys. Furthermore, the percentage of force tuned units in F5 increased during the memory epoch from 8 to 24% in monkey B (Fig. 4e) and from 18 to 27% in monkey S (Fig. 4j), whereas in AIP it only increased from 3 to 6% in monkey B (Fig. 4d) and even decreased from 19 to 10% in monkey S (Fig. 4i). These differences demonstrate a much more prominent role of F5 for the planning of grasp force.

M1 units were recorded only from monkey B (Fig. 4c,f). As expected^17,18,20,21,27^, many units in M1 were tuned for grip type and grip force during movement. However, even though the peak percentage of grip type tuned units was higher in M1 (72%) than in AIP (52%) and F5 (46%), the peak percentage of force tuned units in M1 during the hold epoch (41%) was only slightly higher than in AIP (31%) and similar to F5 (40%). During the cue and memory epoch, the peak percentage of force tuning was lower in M1 (10%) than in F5 of monkey B (15%) and monkey S (27%) and in AIP of monkey S (22%), but higher than in AIP of monkey B (5%), suggesting only a minor role of M1 for force planning. Finally, the interaction effect during the hold epoch was much stronger in M1 than in AIP and F5, and virtually absent before movement onset, suggesting a stronger dependence, or interaction, of force coding in M1 on grip type than in AIP and F5.

The different colors in Fig. 4a–c,g,h show a variety of which force condition evoked the highest firing rate over time. In each brain area, units can be found that are more strongly active in the low force than the high force condition (e.g., Fig. 3b,d), even though high force always evoked more muscle activity (Fig. 2). This indicates a more complex coding of grasp force than a simple increase of neural activity with force^24^.

In additional checks we tested whether the results were affected by the memory delay time (which is negatively correlated with reaction time) and whether single-units responded differently from multi-units. In neither case did we observe major differences in the response patterns, suggesting a robust result.

Together, the presented population results provide an overview of response patterns of individual units and suggest an important role of AIP, F5, and M1 in grasp force control and of AIP and F5 in grasp force planning. However, this kind of analysis largely ignores complex responses during the course of a trial that are visible in individual PSTHs (Fig. 3). While tuning analysis provides a useful overview of an area’s involvement in a task, it does not allow a direct attribution of neural population variance to specific task parameters. In the next section, we therefore utilize a novel dimensionality reduction technique to decompose neural population activity into task-specific latent dimensions that capture most of the neural variance.

### Demixed principal component analysis

To disentangle neural population activity into task-relevant dimensions, we applied a novel dimensionality reduction technique called demixed principal component analysis^28^ (dPCA, see Methods) on the best-channel sets of each area. Like principal component analysis (PCA), this method extracts common components that seek to explain neural population variance. However, in contrast to PCA, dPCA also takes information about the task conditions into consideration.

**Figure 5:**
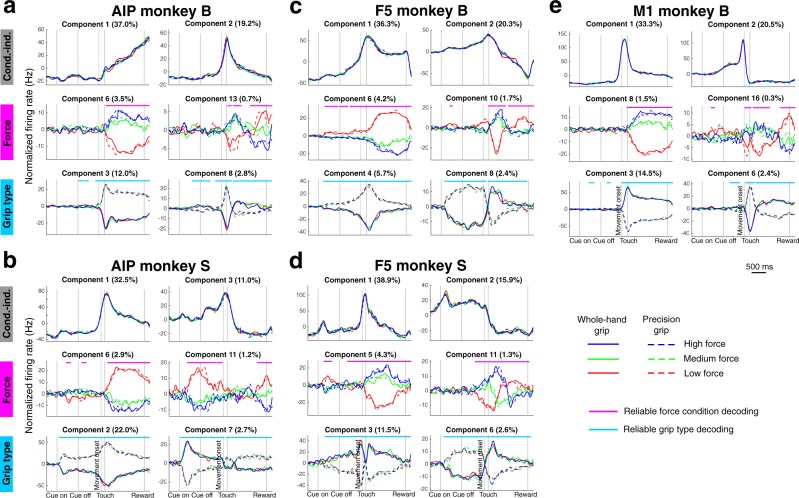
Demixed principal components. Panels a–e show dPCA analysis of the neural population separately for each cortical area and monkey. Each panel depicts the two largest demixed principal components for which variance is mainly attributable to the condition-independent factors (top row), grasp force (second row), and grip type (third row). Red lines: firing rate in low force condition; green lines: medium force condition; blue lines: the high force condition. Solid lines: whole-hand grips; dashed lines: precision grips. Horizontal lines in the second (magenta) and third row (cyan) indicate time intervals when the task condition (force or grip type) could be decoded reliably. X-axis, alignments, epochs, and scale as in Fig. 2.

Figure 5 shows the two largest demixed principal components (dPCs) from each recorded brain area that are mainly affected by grip type, force, or changes over time (condition-independent). The largest component was always condition-independent and explained 32–39% of the neural variance. The second largest component was generally also condition-independent (only in AIP of monkey S this was a grip type component) and explained 16–22% of the variance. The two largest components of each area therefore explained more than half of the total neural variance (53–57%).

To measure whether the differences in firing rate between conditions were significant, we calculated when the respective trial parameters could be reliably extracted from pseudo-single-trial activity by using all recorded neurons (indicated with a magenta or cyan line on top of force and grip type panels, respectively). In Fig. 5, we see a significant difference between force conditions in each brain area during movement execution. However, a significant population force effect, before movement onset, was only observed in F5 of both animals and AIP of monkey S. Similar to the population tuning results (Fig. 4), this suggests a role of F5, but presumably not of M1, in grasp force planning, while the role of AIP remains unclear on the basis of our data.

Significant differences in firing rate for the grip type components occurred before and after movement onset in F5 of both monkeys and AIP of monkey S. Also, in AIP and M1 of monkey B, there are some differences before movement onset, but these differences were not significant for the entire cue and memory epoch, as was the case for AIP of monkey S and area F5 of both animals.

The time course of population activity looked different for the largest grip components than for the largest force components. The firing rate difference between the force conditions was highest in the middle of the hold epoch, while the highest difference between the grip conditions was at the moment of touch (or shortly before touch in case of F5 of monkey S, Fig. 5d). These findings fit well with how the task had to be executed; first the grip needed to be formed, then, after touching the handle, the correct amount of force needed to be applied.

Similar to force, EMG, and single-unit responses (Figs 2 and 3), Fig. 5 also shows that it is the low force condition that first separates from other conditions, while medium and high force separate later, more than 150 ms after object touch. This higher similarity of neural activity between the medium and high force condition could be related to the monkey’s strategy to complete the task, e.g., if monkeys see medium and high force trials as trials where they have to put effort in reaching a certain force level, while low force is easily obtained by not using too much force. Alternatively, it could be related to the higher similarity of EMG activity in the medium and high force vs. the low force condition (see Fig. 2).

**Figure 6:**
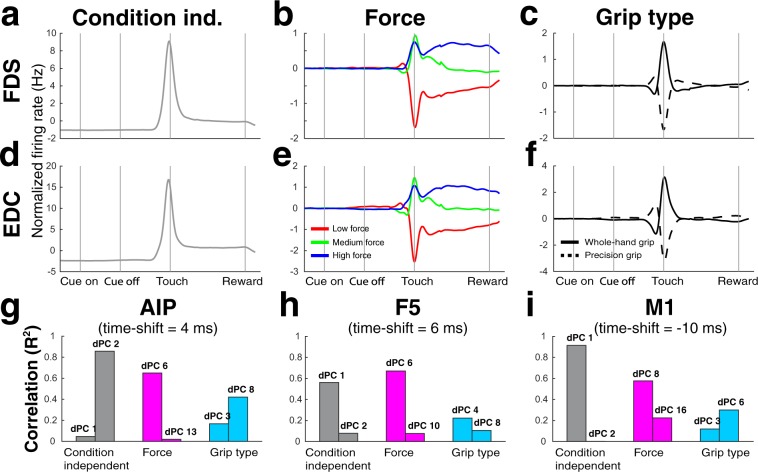
EMG marginalizations. (**a**) Condition-independent marginalization of the EMG of the *flexor digitorum superficialis* (FDS) muscle. (**b**) Marginalizations of the FDS EMG for the low force (red), medium force (green), and high force condition (blue). (**c**) Marginalizations of the FDS EMG for the whole-hand (solid line) and precision grip condition (dashed line). (**d**–**f**) Same as A-C, but for the EMG of the *extensor digitorum communis* (EDC) muscle. X-axis, alignments, epochs and scale in A-F as in Fig. 2. (**g**–**i**) Correlations between the condition-independent, force, and grip type marginalizations of the averaged FDS and EDC EMG with the corresponding first and second dPC of the AIP, F5, and M1 neural population. The time shift for each area was optimized to yield the highest correlation (R^2^-value) between the EMG signal and the first condition-independent component (for AIP: second component), and then used to calculate all other correlations for that area.

These latent variables represent features that are not apparent at the level of individual neurons^23^ and one of the features they could potentially represent, is the activity of muscles in the contralateral hand^29^, as recently found by Gallego *et al*.^24^. We therefore compared the dPCs of monkey B (Fig. 5a,c,e) to a corresponding decomposition of the EMG signals of the *extensor digitorum communis* (EDC) and *flexor digitorum superficialis* (FDS) (see Methods) (Fig. 6a–f).

We can immediately see large similarities between these EMG marginalizations and some dPCs (typically the largest dPC). To quantify how similar they are, we correlated the corresponding averages of the FDS and EDC marginalizations as shown in Fig. 6a–f with the two largest dPCs per factor (time, force, and grip type) of monkey B. Figure 6g–i show the R^2^-values of these correlations for areas AIP, F5, and M1, respectively. High correlation values were found for the condition-independent components (AIP 0.86, F5: 0.56, M1: 0.91) and force components (AIP: 0.65, F5: 0.67, M1: 0.58), while correlations were relatively low for grip type components (AIP: 0.42, F5: 0.22, M1: 0.30). This indicates that a part of the fronto-parietal grasping network population activity resembles contralateral forearm muscle activity, which is more pronounced for coordinating condition-independent factors (e.g. reaching) or grasp force, but less for coordination of grip type. Latent variables that are poorly correlated with the EMG signal may not clearly describe movement output, but could represent internal processes that help compose the output signals^29^.

Correlations between EMG and neural activity could reflect a causal influence of cortical activity driving the muscles or, vice versa, a feedback response from muscle activity in cortex. We therefore correlated EMG marginalizations and dPCA components with variable time-shifts. Time shift values with maximal correlation values suggest in M1 that neural signals precede forearm EMG activity by 10 ms, whereas neural signals of AIP and F5 lagged EMG activity by about 4–6 ms. However, these correlations are not conclusive and further research is required to better understand the role of these brain areas for driving or sensing muscle activity.

Together, dPCA provided an overview of the common components of AIP, F5, and M1 population activity with respect to a delayed grasping task. All cortical areas had latent variables that were dominated by the effect of grasp force, which was present in all areas after movement onset, and prior to movement onset in F5 of both monkeys and AIP of monkey S. Finally, major condition-independent and grasp force dPCA components were highly correlated with the corresponding EMG marginalizations. These correlations became only apparent at the population level.

### Explained variance

**Figure 7:**
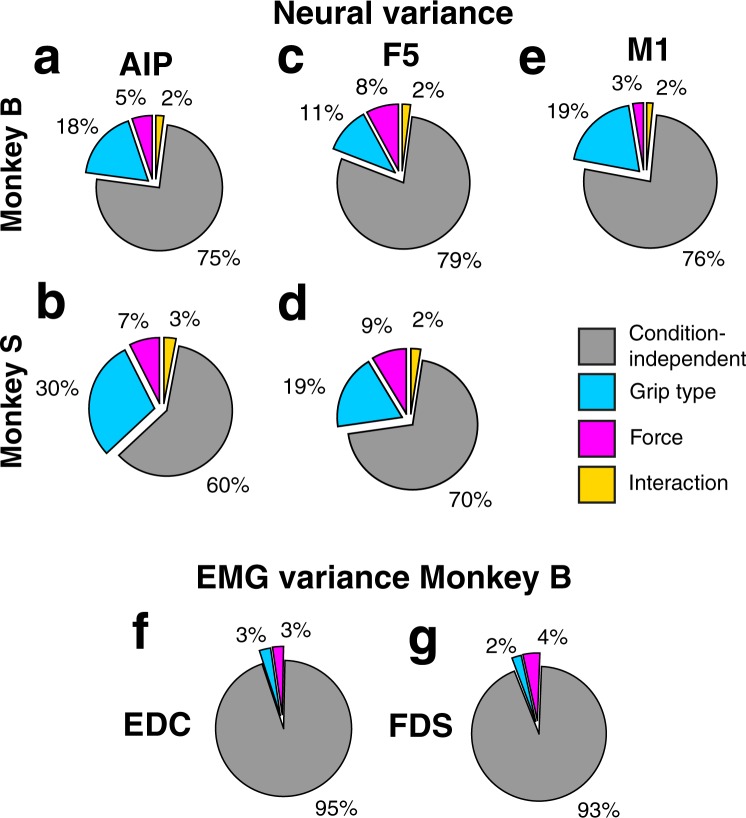
Percentage of explained variance per condition. Variance explained by condition-independent (grey), force (magenta), grip type (cyan), and interaction (yellow) components. (**a**,**c**,**e**) Explained variance of AIP, F5, and M1 of monkey B. (**b**,**d**) Explained variance of AIP and F5 of monkey S. (**f**,**g**) Explained variance of EDC and FDS muscle signal of monkey B.

To quantify the effect of each task parameter on the neural variance, we calculated the percentage of neural variance explained by the condition-independent, grip type, force, and interaction dPCs (Fig. 7a–e). In addition, we calculated the explained variance for the FDS and EDC EMG signals (Fig. 7f,g). For all brain areas, condition-independent parameters explained most of the variance (60–79%), similar to what was found in other neurophysiological studies^28,30^. Furthermore, EMG variance was explained mostly by condition-independent parameters (93–95%). These findings are important to note, because, even though they contribute to most of the variance, condition-independent parameters are usually ignored in many more conventional analysis methods like, for example, population tuning (Fig. 4).

Grasp force-dependent dPCA components explained a surprisingly similar percentage of variance in the brain areas (3–9%) as the corresponding EMG marginalizations (3–4%), thereby demonstrating that these percentages are not insignificant, or at least as significant as the effect of grasp force on the EMG signal. Highest percentages of explained variance were found in F5 (8–9%), followed by AIP (5–7%) and M1 (3%). Variance explained by grip type, in comparison, clearly explained more of the neural variance (11–29%) than of the EMG variance (2–3%). This might point to a more complex or indirect, neural processing of grip type in these areas, at least in comparison to grip force.

Figure 7a-E also shows the percentage of variance explained by the interaction between grip type and force (for the muscle signal this was below 0.5% and is therefore not visible in Fig. 7f,g). For each brain area and muscle, the amount of variance explained by interaction (0–3%) is smaller than that of individual conditions (3–29%). This suggests that information about grip type and force can be largely extracted separately.

Together, neural variance of AIP, F5, and M1 as well as EMG variance of FDS and EDC muscle activity were similarly affected by the condition-independent, grip type-dependent, and grasp force-dependent components or marginalizations. Especially the relative contribution of grasp force to variance was similar between neural and EMG signals. Among the brain areas, F5 variance was most strongly affected by grasp force. Finally, the low percentage explained variance by interaction components showed that information about grip type and force are largely separable in the neural population of AIP, F5, and M1, similar to the muscle signal.

## Discussion

We investigated how single- and multi-units in the macaque brain areas AIP, F5, and M1 represent grasp force during grasp planning and execution. Interestingly, neural populations of all three areas showed coding of grasp force, thereby demonstrating for the first time neural grasp force coding in area AIP. Grasp force modulation was strongest during grasp execution and holding in all three areas, but also present during cue and memory periods, suggesting the presence of grasp force planning. Grasp force coding prior to movement onset was strongest in F5 and weakest in M1. For AIP it differed between animals: in monkey S it was about as strong as in F5, whereas it was almost absent in monkey B. These findings suggest F5 to be most important for grasp force planning.

By employing demixed principal component analysis (dPCA), we could extract latent variables dominated by the effect of time (condition-independent), grip type, or grip force. Differences between force conditions could be reliably extracted during grasp execution from force components of all areas, whereas before movement initiation this was restricted to F5, and including area AIP in case of monkey S. Interestingly, a specific subset of dPCs, in particular time and force, of all three areas were strongly correlated with the corresponding marginalizations of the forearm EMG signals, while other dPCs were not. This division of neural activity was only apparent at the population level, showing that parts of the same neural population activity could directly reflect muscle activity.

In all three brain areas and both forearm muscles, condition-independent parameters explained most of the variance, largely reflecting the overall task timing and common reaching components of the task. Grip type also explained a high percentage of variance in the dPCA components of neural activity, confirming the strong representation of grip type in the fronto-parietal grasping network of AIP, F5, and M1^7,8,10–15^. Importantly, grasp force explained a similar percentage of variance in AIP, F5, and M1, suggesting a representation of grasp force spanning these areas.

### Visual, haptic, or movement related representation

Neurons in F5 and especially in AIP are known to be selective for visual signals as well as movement planning and execution^26^, which makes activity during cue and memory period difficult to interpret. In case of a visual representation, neural activity should resemble the visual cues, which were equally spaced for low, medium and high force (Fig. 1). While, in case of a movement related representation, neural activity should resemble the EMG signals, which were much more similar for the medium and high force conditions than for the low force condition (Fig. 2). In our findings, neural population activity during cue and planning strongly resembled EMG activity for the three force conditions (Fig. 5), suggesting neural activity during these periods to be predominantly movement related.

Neural signals during grasping and holding could also be affected by visual and haptic continuous feedback of the amount of applied grasp force that the monkeys received while touching the handle. As shown before, activity in F5, M1, and especially AIP is affected by sensory (visual or haptic) information^26,31,32^. Presumably, neural signals during grasping and holding are a combination of feed-forward and feedback information about grasp force. Our correlation analysis between neural and EMG marginalizations showed maximal values for M1 activity shortly preceding muscle activity, and for F5 and AIP activity briefly following muscle activity, suggesting that M1 neurons represent feed-forward information, while AIP and F5 neurons rather encode feedback signals. However, more research is needed to further investigate this relationship.

### Grasp force coding in AIP

The parietal cortex was originally thought to strictly code kinematics and not kinetics^33,34^. However, this idea has been challenged by multiple recent studies that have demonstrated force modulation in the parietal cortex^35–40^. Our results provide evidence of force coding in parietal cortex, in agreement with these studies. Furthermore, we have demonstrated that the percentage of force-tuned units as well as the variance explained by force in AIP is of similar magnitude in F5 and M1, and only slightly weaker than grip type coding.

The role of AIP in grasp force planning remains unclear, since we found grip force representation in monkey S, but hardly in AIP of monkey B (Figs 4, 5). Visual conditions like illumination of the handle were identical for both monkeys and can therefore not explain the observed differences. However, they might be caused by the fact that we recorded from AIP in the right hemisphere of monkey B and in the left hemisphere of monkey S. Although, to our knowledge, no lateralized differences are known for macaque AIP, several studies in humans have characterized the human homologue of AIP in the left hemisphere as more involved in dynamic force control, whereas in the right hemisphere it was more involved with static force control^36,38,39,41^. Also, grasp force planning in humans could only be impaired by inactivating the left-sided AIP, but not the right AIP, independent of which hand was used^35^. These findings raise the possibility that grasp force planning is also lateralized in macaque AIP, but more research is necessary to test this hypothesis.

Finally, another reason for differences between animals could be the electrode locations. Since F5 and especially AIP are relatively small regions in the macaque cortex, anatomical differences between animals and variation in recording quality can be an explanation for differences between monkeys.

### Grasp force coding in F5 and M1

Several studies have demonstrated the representation of grasp force in F5 and M1^17,18,20–22,27^. In one study no correlation was found between the weight of an object and ventral premotor activity during lifting with a precision grip^42^. However, grip force did not vary with the weight of the lifted object in this study. Our finding of a stronger grasp force representation in F5 than in M1 seems to contradict earlier findings^22^. One possible explanation for this could be differences in task design. Their study did not include a reaching phase, since monkeys already held the force transducer in their hand at the start of the trial. It is therefore reasonable to compare their results with the later part of the hold epoch in our task, e.g., the final 500 ms of the hold epoch, where we also found the percentage of tuned units about 10% lower in F5 than in M1. Furthermore, their study did not include a memory period, while we found neurons in F5 strongly modulated during grasp force planning, in contrast to M1 (Figs 4, 5). This suggests that F5 plays an important role for the planning of grasp forces, similar to its well-known role for grip type planning^25,26^.

### Relation between grip type and force

We also investigated how grasp force coding differed between different grip types. In the example units of Fig. 3 we observed that force coding can be different between grip types, but in Fig. 4 we demonstrate that the percentage of units tuned for interaction is for each brain area and at all time points lower than the percentage of units tuned for grasp force, suggesting that most units encode grip force similarly for both grip types. Also, in dPCA, the variance explained by interaction (2–3%) is lower than by grip type (11–29%) or force (3–9%) (Fig. 7), indicating that grip type and force can, for the most part, be extracted independently from each other. Together, these findings suggest that information for grip type and force is mostly coded independent from each other, as has been shown previously for M1 and dorsal premotor cortex^43^.

### Cortical representation of muscle activity

It is still an open question whether movement related activity in cortex resembles muscle activity or higher-level movement parameters^29^. We found subsets of neural population dynamics strongly correlated with muscle activity, while other subsets were not (Fig. 6), well in line with recent findings^24,44^. Intriguingly, muscle activity-related and unrelated subsets became apparent as a result of population level analyses. Population level analyses in turn reveal covariant patterns of neural dynamics, which are all derived from the whole population, suggesting that activity of individual neurons in AIP, F5, and M1 is partially muscle related and partially not. Our results could be of particular importance with respect to this controversy, since our task includes an often neglected, crucial parameter of muscle activity, grasp force. In summary, our results indicate that subsets of the neural population dynamics are indeed reflecting muscle activity, while the same neurons are involved in other processes as well.

## Methods

### Basic procedures

One male (monkey B) and one female (monkey S) rhesus macaque (*Macaca mulatta*, 13.5 and 10.1 kg, respectively) were used in this study. All animal care and experiments with the animals were performed in accordance with German and European law and in agreement with the *Guidelines for the Care and Use of Mammals in Neuroscience and Behavioral Research*^45^ and the *NC3Rs Guidelines*^46^, and were approved by the Animal Welfare Division of the Office for Consumer Protection and Food Safety of the State of Lower Saxony, Germany.

Both monkeys were habituated to comfortably sit in a primate chair with the head fixed. Before starting the task, they were placed in front of a grasping handle at a horizontal distance of ~26 cm in a dark room. One or two capacitive sensors (Model EC3016NPAPL; Carlo Gavazzi) located at the level of the monkey’s mid-torso were placed in front of monkey B and S, respectively, and served as hand rest position (handrest buttons). Monkey S performed the task with her right hand and was trained to keep her left hand on the handrest button for the duration of the trial. Monkey B performed the task with his left hand and his right arm was placed in a long tube, preventing it from interacting with the handle. Visual cues were projected from a TFT screen (CTF846-A; Screen size: 8″ digital; Resolution 800 × 600; Refresh rate: 75 Hz) onto the center of the handle via a half mirror. The TFT screen was masked so that a direct view of the image was impossible. Eye movements were tracked with an infrared optical eye tracker (AA-ETL-200; ISCAN, Woburn, USA) that was calibrated at the start of each session. Eye tracking and the behavioral task were controlled with custom-written software implemented in LabView Realtime (National Instruments, Austin, TX, USA) with a temporal resolution of 1 ms. Monkey behavior was continuously monitored via an infrared camera during task performance.

### Task paradigm

Monkeys were instructed by visual cues about how and when to grasp (Fig. 1A). A trial was initiated by placing the acting hand on the handrest button where it had to remain until the Go signal was given. A red dot was projected at the same location as the grasping handle and functioned as eye fixation target. Animals were required to fixate this dot for the entire trial duration to prevent eye movements from confounding the neural signals^47^.

After fixating for 400–500 ms (Fixation epoch), two spotlights illuminated the grasping handle for 800 ms and an instruction cue appeared on the left or right side of the fixation dot during this time (cue epoch). The location of this cue instructed the animals which grip type to perform. A cue on the left side instructed a whole-hand grip (opposition of fingers and palm), while a cue on the right side instructed a precision grip (opposition of the tips of the thumb and index finger). As illustrated in Fig. 1A, the cue consisted of a green disk and a grey bar in front. A white area within this grey bar, named force target, indicated how much force the monkey had to apply: when the force target was at the bottom of the bar, 0–5 N was required (low force), when this was one level higher, 5–9 N was required (medium force), and when the target was in the middle of the bar, 9–12 N was required (high force). Note that for the low force condition a small amount of force was required to activate the two touch sensors. Force values were identical for whole-hand and precision grip trials. The selected force range was based on the amount of force the monkeys naturally applied to the handle before being trained on the force cues.

After the cue epoch, instruction cues and illumination were turned off. The monkeys were required to memorize the instruction and continue fixating for 500–700 ms (memory epoch). At the end of the memory epoch, the fixation dot briefly blinked to instruct the monkeys to reach and grasp the target (Go signal). Movement initiation was detected when a monkey lifted its grasping hand from the handrest button. Note that lifting of the hand from the handrest button prior to the Go signal resulted in an immediate abortion of the trial. In order to prevent the animals from predicting the Go signal, in addition to the variable memory time, trials were aborted in which the animals lifted of their grasping hand from the handrest button within 100 ms after the Go signal. This additional control was introduced at an early stage of the task training resulting in a neglectable number of premature movements during the recording sessions.

Handle touch (Touch) was defined in case of a whole-hand grip as the moment when the hand interrupted the infrared light barrier of the handle, or in case of a precision grip when the thumb and index finger triggered two touch sensors on the side of the handle. After Touch, the force cue would reappear as well as the red slider bar indicating the applied grasp force. Animals had to apply sufficient grasp force to bring the red slider into the white area and keep it there for one second (hold epoch). In case animals left the force target early the timer restarted. However, this only happened in very few cases during the recording sessions. Finally, all correctly executed trials were rewarded with a liquid reward (reward). The handle was only visible during the cue epoch. All grasping and holding actions were performed in darkness so that neural modulations could not be caused by the visual observation of the setup or the grasping action.

Since pushing or pulling of the handle also increases the air pressure in the tube and therefore can lead to potential artifacts of grasp force measurements, we restricted the monkeys to not push or pull the handle with a force that was greater than necessary to achieve the required pressure force. This allowed us to measure grasping force reliably. See supplementary information for more details about the force sensing handle.

The task consisted of six conditions, which were all combinations of both grip types and three force levels, resulting in: whole-hand low force (WLF), whole-hand medium force (WMF), whole-hand high force (WHF), precision low force (PLF), precision medium force (PMF), and precision high force (PHF). Trials were presented in pseudo-random order. Trials were initially drawn from a pool of 30 trials, containing 5 copies of the six conditions. In case the monkey successfully performed a trial of a certain condition, it was removed from the pool. The pool was refilled with a copy of each of the six conditions whenever the pool contained less than 25 trials (i.e. the pool size always varied between 25 to 30 trials). In case the monkey unsuccessfully performed a trial of a certain condition, the trial of that condition stayed in the pool, making it impossible for the monkeys to selectively skip certain conditions. Hence, when one condition was not performed successfully as often as others, it would occur more frequently, but monkeys could not predict which condition appears in the next trial.

### Behavioral data analysis

To measure how well the monkeys performed the task, we calculated two different measures of performance: 1. Percentage of successful trials initiated after cue epoch onset; 2. Percentage of successful trials initiated after Go signal. The first measure indicates the condition preference, since the monkeys broke eye fixation more often for conditions they preferred less. The second measure indicates how well the monkeys could perform the task, because the monkeys now tried to complete the task, but were not always successful. Only successful trials were used in further analysis.

Reaction time (RT) was calculated as the time between Go signal and Movement initiation. Movement time (MT) was the time between Movement initiation and handle touch. Acquisition time (AT) was the time between handle touch and when the required force level was achieved. Note that due to the sensitivity of the force sensor, an increase in force was already detected before the touch sensors detected a handle touch. The monkey could therefore already apply significant force to complete the low force condition at the moment of handle touch and the acquisition time could therefore be close to zero.

Trials with unusually long response times (RT > 0.5 s, MT > 0.35 s, and AT > 1.5 s) and cases where the monkey touched sensors more than once were excluded from behavioral, EMG, and neural analyses (14% of trials from monkey B and 9% of trials from monkey S). Response time thresholds were set based on visual inspection of the response time distributions of several sessions from both monkeys.

### Grasp force and electromyography analysis

We recorded grasp force in all 11 sessions of monkey B and surface electromyography (EMG) signals in 2 sessions. Grasp force of monkey S (5 sessions) was recorded online, but not stored for later analyses. Force signals were smoothed with a Gaussian kernel (σ = 10 ms, binsize = 2.5σ) to reduce noise. Surface EMG activity was recorded from the ventral and dorsal lower arm using self-adhesive electrodes and the Neurolog amplifier (NL844 and NL820; Digitimer). EMG activity originated primarily from the *flexor digitorum superficialis* (FDS) muscle and the *extensor digitorum communis* (EDC) muscle. Other muscles, including biceps and triceps, were also explored, but lower arm EMG was best in distinguishing the task conditions and therefore selected. EMG signals were band-pass filtered (25–250 Hz, 6^th^ order Butterworth), rectified, smoothed (Gaussian, σ = 10 ms, binsize = 2.5σ), and normalized by dividing it by the activity during the Fixation epoch^48^. Note that only trial averaged EMG signals were used for all analyses. Due to their high degree of similarity between sessions, we recorded EMG signals only from 2 sessions.

### Neural recordings

Recordings were made in an electrically shielded room to prevent electromagnetic noise. Signals from the implanted 32-channel arrays (see Supplementary Information) were amplified and digitally stored using a 128-channel recording system (Cerebus; Blackrock Microsystems; sampling rate 30 kS/s; 0.3–7500 Hz hardware filter). Neural recordings of all 4 arrays (2 in AIP and 2 in F5; 128 channels total) from 5 sessions from monkey S were processed and analyzed. Neural recordings from monkey B were also 128 channels in total, but they could either be recorded from 2 arrays in AIP and 2 in F5 (7 sessions), 2 in AIP and 2 in M1 (2 sessions), 2 in F5 and 2 in M1 (1 session), or 2 in AIP, 1 in F5, and 1 in M1 (1 session). In total, 11 sessions from monkey B were processed and analyzed.

In offline processing, data were median filtered (window length: 3.33 ms) and the result subtracted from the raw signals. Afterwards, the signal was low-pass filtered with a non-causal Butterworth filter (5000 Hz; 4^th^ order). Furthermore, to eliminate common noise-sources, principal component artifact cancellation was applied for all electrodes of each array, which has previously been described^49,50^. To ensure that no individual channels were eliminated, all PCA dimensions with a coefficient exceeding 0.36 (with respect to normalized data) were retained.

### Peri-stimulus time histograms

Spiking events of each unit were downsampled to 1 kHz and smoothed with a Gaussian window (σ = 50 ms). Smoothed spike events were aligned to cue onset (400 ms before and 1300 ms after), touch (500 ms before and 500 ms after), and reward onset (1000 ms before and 200 ms after). Peri-stimulus time histograms (PSTHs) were computed by averaging the spike events across trials for each alignment. These three PSTHs were then combined to produce a smoothed, continuous signal.

However, PSTHs aligned at different events can contain the same information. For example, when acquisition time (AT) is close to 0 ms (true for most low force trials), the 500 ms after touch contains the same information as the 1000–500 ms before reward onset. Therefore, when the median AT was less than 500 ms, we did not show the part of the plot from 500 ms after touch until median AT + 500 ms before reward. Only for the statistical tests described below, we interpolated the activity for this overlapping period.

### Cluster-based permutation test

To test for significant differences in firing rate between conditions at every time point and to adequately deal with the multiple comparison problem, we used a cluster-based permutation test^50^. In short, we first applied a two-way ANOVA in 10 ms steps along the PSTH and selected all time points with p < 0.01 (this is not the final testing statistic). In the next step, all adjacent time points with p < 0.01 were combined as a cluster and their F-values summed. This was done separately for the p-values of the grip type main effect, force main effect, and interaction effect. We then created 1000 shuffled data sets by randomly rearranging the condition labels of trials of the data and applied the same procedure on these shuffled data sets. In each shuffled data set, the cluster with the highest summed F-value was selected to create a distribution of F-values (null distribution). Summed F-values in the recorded data were then considered significant if they exceeded the 99%-percentile in the null distribution (i.e., α-level = 0.01). An α-level of 0.01, instead of 0.05, was used to decrease the false positive rate to better emphasize the main effect.

### Demixed principal component analysis

To get an overview of the population activity from the hundreds of neurons we recorded simultaneously, one could use principal component analysis (PCA) to extract linear combinations of the spiking activity population responses (PSTHs) that can explain a large majority of the observed variance. However, since individual units can show a mixed selectivity for different task parameters, standard PCA does not provide much information about the influence of specific task parameters on neural variance. It is therefore important to ‘demix’ this selectivity to understand this relation.

To do this, we applied a novel dimensionality reduction technique called demixed principal component analysis (dPCA)^28,30^ on the data, using freely available code: http://github.com/machenslab/dPCA. Like standard principal component analysis, dPCA extracts the components of a high-dimensional dataset that describes most of the variance and calculates how much variance is explained by each component. However, unlike standard PCA, dPCA uses information about the task conditions (i.e. grip type, force, or interaction) to calculate the percentage of variance explained by each task condition and by condition-independent changes over time. Condition-independent components reflect dynamical changes of the population activity over the time course of the trial which are similar for all conditions. It then creates components that are primarily affected by a specific task condition. In addition, this toolbox uses a linear classifier (stratified Monte Carlo leave-group-out cross-validation) to reveal at which time points the conditions are significantly different from each other.

For each brain area, a five-dimensional matrix of the firing rate was constructed: one dimension for the units in the best-channel set of that brain area (see Supplementary Information), one dimension for the three force conditions, one dimension for the two grip types, one dimension for the down-sampled time points (390 time points with steps of 10 ms), and one dimension for the trials. This five-dimensional matrix was used as input for the toolbox, but for calculating the demixed principal components (dPCs), trial averages were used (i.e. a four-dimensional matrix), which was therefore not affected by the intrinsic trial-by trial variability of the neurons.

The toolbox was used with the following parameters: the first 30 components were calculated, the number of repetitions used for optimal lambda calculation was 10, the number of iterations for cross-validation was 100, and the number of shuffles used to compute the Monte Carlo chance distribution was set to 100, as described previously^28^. Time periods when the actual classification accuracy exceeded all 100 shuffled decoding accuracies in at least 10 consecutive time bins were marked with colored lines on top of the figures showing the dPCs. Note that statistical significances of conditional tuning of individual dPCs were dependent on the intrinsic neuronal variability, since they were based on a single trial shuffling procedure.

### EMG marginalizations

To compare the dPCA components found in the neural population of the best-channel set with the trial- and session-averaged EMG signal, we decomposed the EMG signals also into averaged signals (marginalization) of the condition-independent component, force condition, and grip type condition. Note that we did not apply dPCA to EMG signals, since only 4 muscles were recorded which is not sufficient for dPCA. This was done separately for each muscle in the same way as dPCA^28^:

$$\bar{x}={\langle x\rangle }_{tfg}$$

$${\bar{x}}_{t}={\langle x-\bar{x}\rangle }_{fg}$$

$${\bar{x}}_{f}={\langle x-\bar{x}\rangle }_{tg}$$

$${\bar{x}}_{g}={\langle x-\bar{x}\rangle }_{tf}$$

$${\bar{x}}_{tf}={\langle x-\bar{x}-{\bar{x}}_{t}-{\bar{x}}_{f}-{\bar{x}}_{g}\rangle }_{g}$$

$${\bar{x}}_{tg}={\langle x-\bar{x}-{\bar{x}}_{t}-{\bar{x}}_{f}-{\bar{x}}_{g}\rangle }_{f}$$

$${\bar{x}}_{fg}={\langle x-\bar{x}-{\bar{x}}_{t}-{\bar{x}}_{f}-{\bar{x}}_{g}\rangle }_{t}$$

$${\bar{x}}_{tfg}=\langle x-\bar{x}-{\bar{x}}_{t}-{\bar{x}}_{f}-{\bar{x}}_{g}-{\bar{x}}_{tf}-{\bar{x}}_{tg}-{\bar{x}}_{fg}\rangle $$

With $\bar{x}$ being the overall mean firing rate of the EMG signal. Angular brackets denote the average over time (t), force (f), and/or grip type (g). The condition-independent marginalization (${\bar{x}}_{t})$ is the average signal of every time point minus the overall mean firing rate. The force marginalization is the sum of the time-independent marginalization $({\bar{x}}_{f})$ and the time-force interaction $({\bar{x}}_{tf})$, because we expect all EMG marginalizations, like the neural components, to change with time. Likewise, the grip type marginalization is the sum of ${\bar{x}}_{g}$ and ${\bar{x}}_{tg}$. The interaction marginalization consists of the sum of ${\bar{x}}_{fg}$ and ${\bar{x}}_{tfg}$.

These EMG marginalizations (condition-independent, force, and grip type) were then correlated with the two largest dPCs of the respective neural marginalization. The three force conditions of the EMG marginalizations were concatenated in the same order as the force conditions of the dPCs were concatenated to compute one correlation value for force. The same was done for the two grip type conditions.

In order to compensate for possible feedforward and feedback delays between the neural and the muscle signals, we calculated R^2^-values with several time-shifts, ranging from −500 to 500 ms. The time shift that yielded the highest R^2^-value when correlating the largest neural component with the EMG signal was chosen to calculate all R^2^-values for that brain area. Only for AIP we chose the second-largest instead of the largest component, because it was correlated much more strongly (R^2^ = 0.86) with the EMG signal, than the largest component (R^2^ = 0.05).

## Electronic supplementary material

Supplementary Information

## Data Availability

The datasets recorded and analyzed for the current study are available from the corresponding author upon reasonable request.
