# Supplementary material for: Neural coding of intended and executed grasp force in macaque areas AIP, F5, and M1
Source: Sci Rep. 2018 Dec 20;8:17985. doi: 10.1038/s41598-018-35488-z (PMC6301980; doi:10.1038/s41598-018-35488-z)
Supplement: Supplementary file 1 — Supplementary Information [file 41598_2018_35488_MOESM1_ESM.pdf]

Neural coding of intended and executed grasp force in macaque areas AIP, F5, and M1

Rijk W. Intveld[1], Benjamin Dann[1], Jonathan A. Michaels[1,2], Hansjörg Scherberger[1,3,†]

[1]Deutsches Primatenzentrum GmbH, Kellnerweg 4, 37077 Göttingen, Germany

[2]Department of Electrical Engineering, Stanford University, Stanford, CA 94305, USA

[3]Faculty of Biology and Psychology, University of Göttingen, 37073 Göttingen, Germany

[†]Corresponding author. Email: hscherberger@dpz.eu

Supplementary table

Supplementary table 1. Number of single-, ambiguous-, and multi-units units per average session and in the best-channel set.

Only sessions with data from two arrays per brain area were used to calculate the average number of units in that brain area. When no units were recorded from an area, it is labeled as NR (Not Recorded).

**AIP**	**Monkey B, average session**	**Monkey B, best-channel set**	**Monkey S, average session**	**Monkey S, best-channel set**
**Single-units**	11.2	47	24.0	56
**Ambiguous-units**	3.1	10	6.2	11
**Multi-units**	14.8	34	36.8	45
**Total**	29.1	91	67.0	112
**F5**	**Monkey B, average session**	**Monkey B, best-channel set**	**Monkey S, average session**	**Monkey S, best-channel set**
**Single-units**	14.9	62	29.6	57
**Ambiguous-units**	4.0	9	5.6	7
**Multi-units**	27.4	55	28.0	29
**Total**	46.3	126	63.2	93
**M1**	**Monkey B, average session**	**Monkey B, best-channel set**	**Monkey S, average session**	**Monkey S, best-channel set**
**Single-units**	18.0	40	NR	NR
**Ambiguous-units**	3.0	8	NR	NR
**Multi-units**	30.7	39	NR	NR
**Total**	51.7	87	NR	NR

Supplementary methods

Surgery and implantation

Surgery and implantation procedures have been described before[1,2]. A head post (titanium cylinder; diameter, 18 mm) was implanted to allow for eye position tracking and to prevent head-movement related noise in the neural signal. Array implantation followed several months after the animals could adequately perform the grasping task with eye fixation.

An MRI scan was done to locate anatomical landmarks like the arcuate, central and intraparietal sulci (for area F5, M1 and AIP, respectively) to guide array implantation. For this, monkeys were sedated (e.g., 10 mg/kg ketamine and 0.5 mg/kg xylazine, i.m.) and placed in the scanner (GE Healthcare 1.5T or Siemens Trio 3T) in a prone position. Analysis of T1-weighted volumetric images of the brain and skull was done as described previously[3]. The arcuate sulcus of monkey S did not present a spur, but a small indentation in the posterior bank of the arcuate sulcus, about 2 mm medial to the knee, which was treated as a spur. FMAs were placed lateral to that mark.

Monkey S and monkey B were implanted with 2 floating microelectrode arrays (FMAs; MicroProbe for Life Science) in area F5 and 2 in AIP (Figure 1B and C). Monkey B was also implanted with 2 additional FMAs in area M1 (Figure 1B). All arrays were implanted in the hemisphere contralateral to the arm used in the experiment (monkey S: left hemisphere, monkey B: right hemisphere). FMAs consisted of 32 non-moveable monopolar platinum-iridium electrodes used for recordings, 2 grounds, and 2 reference wires. Electrode impedances at 1 kHz ranged between 300 and 600 kΩ and the lengths of electrodes were between 1.5 and 7.1 mm, with longest electrodes closest to the sulcus. However, since electrodes were placed in the sulcus rather tangentially, we were not able to determine from which cortical layer recordings were made.

All surgical procedures were performed in sterile conditions while the monkeys were anaesthetized (e.g., induction with 10 mg/kg ketamine, i.m., and 0.05 mg/kg atropine, s.c., followed by intubation, 1–2% isoflurane, and analgesia with 0.01 mg/kg buprenorphine). Heart and respiration rate, electrocardiogram, oxygen saturation, and body temperature were monitored continuously. Systemic antibiotics and analgesics were administered for several days after each surgery. Animals were mildly hyperventilated to prevent brain swelling while the dura was open (end-tidal CO_2, ~30 mmHg) and Mannitol was administered as necessary. Training and recording sessions recommenced only after the monkeys were fully recovered from the surgery, which took about 2 weeks.

Force sensing handle

To measure grip type and force simultaneously, we designed a new grasping handle (Figure 1D). This handle was similar to handles that we used in previous experiments[1-6], including an infrared (IR) light barrier located inside of the handle opening to detect a whole-hand grip and two touch sensors

located sideways on the handle to detect precision grips. However, unlike the former handle, the handle used here consisted of an air-filled rubber tube that was connected to an air pressure sensor. Applied grasp force was then measured by comparing the air pressure during grasping to the pressure shortly before the handle was touched.

Air pressure in the tube was set to a value between 190 and 210 kPa before an experiment started, since this provided the most stable grasp force recordings. Over the course of an experiment, the pressure did not change by more than 2 kPa. Force was measured continuously over the course of an experiment for both monkeys and furthermore recorded for monkey B. To be entirely unaffected by changing air pressure over time, the baseline pressure (corresponding to zero force) was reset during the fixation epoch of each trial. When executing the task, monkeys were required to exceed this baseline pressure in the low level condition, and correspondingly higher thresholds in the medium and high force condition.

In addition, the handle was mounted on an S-shaped force sensor (KD24s, ME-Messysteme GmbH) to measure push and pull forces.

Spike sorting

Single- and multi-units were extracted and sorted with a modified version of the offline spike sorter *Wave_clus*[5,7,8]. In a first step, spike waveforms were detected by a median-based threshold procedure and aligned on their maximum absolute value. In a second step, features were estimated by calculating the wavelet decomposition and PCA in addition to all time samples of the original waveforms. In a third step, 17 features with the highest p-value, rejecting the null hypothesis of being from the family of normal distribution, were selected, which potentially allows for a good separation of different units. In a fifth step, superparamagnetic clustering was applied on the selected features[7]. In a sixth step, combinations of the different clusterings were manually selected to best separate the present single- and multi-unit waveforms. In a seventh step, the unassigned waveforms were template-matched, using a linear discriminant analysis (LDA) classifier trained on the assigned waveforms. As a final step, redetection of the different templates was done in order to detect overlaid waveforms[5,9]. For this, band-pass filtered signals, as described above, were convolved with the average templates, from high to low amplitude, serving as an optimal filter for the corresponding waveform. Redetection and resorting was run automatically for each template using a linear discriminate analysis for classifying waveforms. After identifying the preferred waveforms, the shift-corrected template was obtained by up and down sampling and subtracted from the filtered signal of the corresponding channel to reduce interference with the detection of the next template. Waveforms were then again visually inspected and clusters of similar-looking waveforms on the

same channel were merged. This procedure allowed a detection of templates up to an overlap of 0.2 ms.

To determine whether units should be classified as single- or multi-units, each waveform shape and inter-spike interval distribution was evaluated. A unit was classified as single-unit when it met the following four criteria: 1. Waveform clusters clearly separated in the projections of the 17 selected features; 2. Detected spike waveforms spread homogeneously around the mean waveform; 3. Well-known waveform shapes of single-units were present; 4. Inter-spike intervals of 2 ms or less were rare or absent. If a unit was clearly identified as an artifact or had an extremely low spike count (<1000 samples per recording session), it was classified as noise and discarded from further analyses. If most, but not all criteria were met, it was classified as an ambiguous unit. All other cases were classified as multi-units. This classification was only used for selecting example units and for creating the 'best-channel set' (see below). In all other analyzes, single-, ambiguous- and multi-units are treated equally.

Best-channel set

In recordings with permanent implants, there is a high chance to record from the same neuron in different sessions, since the chronically implanted floating electrode arrays remain in the same location over the course of an experiment[10]. However, even when the same neuron is recorded over multiple days, the quality of this recording can still vary due to factors such as external noise, plasticity effects, or micro-motions of the electrodes[11-13]. Because of these variations across recording days, any measurable characteristic of a single-unit, such as waveform shape or spiking frequency, can be different in the next session, making it is very difficult to determine whether two single-units recorded on different days on the same electrode actually originate from the same neuron or not.

In order to combine neural data from all recorded sessions per monkey (11 monkey B, and 5 monkey S) without using the same unit multiple times, we created the 'best-channel set'. This is a virtual dataset that consists of neural data from multiple sessions, but data from every electrode/channel is represented only once. For each channel, we counted for each recording session the number of single-, ambiguous- and multi-units, and selected for each channel the recording session that maximized the number of single-, ambiguous-, and multi-units (in that order). If still unclear, the session was selected with the highest number of spikes in single-unit, ambiguous-unit, or multi-unit group (in that order). Combing all of these best sets for each channel resulted in a "best-channel" data set of 192 channels (AIP: 91 units, F5: 126 units, M1: 87 units) for monkey B and 70 channels for monkey S (AIP: 112 units, F5: 93 units), and was used for all subsequent population analyses.

Session selection

Sessions were selected based on task performance and quality of neural signals. For monkey S, 5 sessions were selected that met these criteria. For monkey B, 6 sessions were selected that were similar to the 5 sessions of monkey S and that were recorded from two F5 and two AIP arrays. In addition, 3 more sessions were added with recordings from two M1 arrays and 2 more sessions with EMG recordings. Together in monkey B, 10 sessions were recorded from AIP, 9 from F5, and 4 from M1. These numbers would suggest that more neural information was collected from monkey B, but since the recordings from monkey S were of higher neuronal yield (neurons per session), it was only by using more sessions of monkey B and using the 'best-channel set' method, that the number of recorded units from both monkeys were comparable.

References

1.Townsend, B. R., Subasi, E. & Scherberger, H. Grasp movement decoding from premotor and parietal cortex. *Journal of Neuroscience* **31,** 14386–14398 (2011).

2.Michaels, J. A., Dann, B., Intveld, R. W. & Scherberger, H. Predicting Reaction Time from the Neural State Space of the Premotor and Parietal Grasping Network. *J Neurosci* **35,** 11415–11432 (2015).

3.Baumann, M. A., Fluet, M. C. & Scherberger, H. Context-Specific Grasp Movement Representation in the Macaque Anterior Intraparietal Area. *Journal of Neuroscience* **29,** 6436–6448 (2009).

4.Lehmann, S. J. & Scherberger, H. Reach and gaze representations in macaque parietal and premotor grasp areas. *Journal of Neuroscience* **33,** 7038–7049 (2013).

5.Dann, B., Michaels, J. A., Schaffelhofer, S. & Scherberger, H. Uniting functional network topology and oscillations in the fronto-parietal single unit network of behaving primates. *Elife* **5,** 2870 (2016).

6.Fluet, M.-C., Baumann, M. A. & Scherberger, H. Context-specific grasp movement representation in macaque ventral premotor cortex. *Journal of Neuroscience* **30,** 15175–15184 (2010).

7.Quiroga, R. Q., Nadasdy, Z. & Ben-Shaul, Y. Unsupervised spike detection and sorting with wavelets and superparamagnetic clustering. *Neural computation* (2004). doi:10.1371/journal.pbio.0000042

8.Kraskov, A., Dancause, N., Quallo, M. M., Shepherd, S. & Lemon, R. N. Corticospinal neurons in macaque ventral premotor cortex with mirror properties: a potential mechanism for action suppression? *Neuron* **64,** 922–930 (2009).

9.Gozani, S. N. & Miller, J. P. Optimal discrimination and classification of neuronal action potential waveforms from multiunit, multichannel recordings using software-based linear filters. *IEEE Trans Biomed Eng* **41,** 358–372 (1994).

10.Dickey, A. S., Suminski, A., Amit, Y. & Hatsopoulos, N. G. Single-unit stability using chronically implanted multielectrode arrays. *Journal of Neurophysiology* **102,** 1331–1339 (2009).

11.Barrese, J. C. *et al.* Failure mode analysis of silicon-based intracortical microelectrode arrays in non-human primates. *J Neural Eng* **10,** 066014 (2013).

12.Barrese, J. C., Aceros, J. & Donoghue, J. P. Scanning electron microscopy of chronically implanted intracortical microelectrode arrays in non-human primates. *J Neural Eng* **13,** 026003 (2016).

13.Lee, H., Bellamkonda, R. V., Sun, W. & Levenston, M. E. Biomechanical analysis of silicon microelectrode-induced strain in the brain. *J Neural Eng* **2,** 81–89 (2005).
